# Optimizing non-invasive radiofrequency hyperthermia treatment for improving drug delivery in 4T1 mouse breast cancer model

**DOI:** 10.1038/srep43961

**Published:** 2017-03-13

**Authors:** Matthew J. Ware, Martyna Krzykawska-Serda, Jason Chak-Shing Ho, Jared Newton, Sarah Suki, Justin Law, Lam Nguyen, Vazrik Keshishian, Maciej Serda, Kimberly Taylor, Steven A. Curley, Stuart J. Corr

**Affiliations:** 1 Baylor College of Medicine, Department of Surgery, Houston, TX, USA; 2 Department Biophysics Faculty of Biochemistry, Biophysics and Biotechnology, Jagiellonian University, Krakow, Poland; 3 Interdepartmental program in Translational Biology and Molecular Medicine, Baylor College of Medicine, Houston, TX, USA; 4 Institute of Chemistry, University of Silesia, Katowice, Poland; 5 Rice University, Department of Chemistry, Houston, TX, USA; 6 Rice University, Department of Mechanical Engineering and Materials Science, Houston, TX, USA; 7 University of Houston, Department of Biomedical Engineering, Houston, TX, USA

## Abstract

Interactions of high-frequency radio waves (RF) with biological tissues are currently being investigated as a therapeutic platform for non-invasive cancer hyperthermia therapy. RF delivers thermal energy into tissues, which increases intra-tumoral drug perfusion and blood-flow. Herein, we describe an optical-based method to optimize the short-term treatment schedules of drug and hyperthermia administration in a 4T1 breast cancer model via RF, with the aim of maximizing drug localization and homogenous distribution within the tumor microenvironment. This method, based on the analysis of fluorescent dyes localized into the tumor, is more time, cost and resource efficient, when compared to current analytical methods for tumor-targeting drug analysis such as HPLC and LC-MS. Alexa-Albumin 647 nm fluorphore was chosen as a surrogate for *nab*-paclitaxel based on its similar molecular weight and albumin driven pharmacokinetics. We found that RF hyperthermia induced a 30–40% increase in Alexa-Albumin into the tumor micro-environment 24 h after treatment when compared to non-heat treated mice. Additionally, we showed that the RF method of delivering hyperthermia to tumors was more localized and uniform across the tumor mass when compared to other methods of heating. Lastly, we provided insight into some of the factors that influence the delivery of RF hyperthermia to tumors.

Breast cancer leads in estimated new cancer cases (29%), and is the second most common cause of cancer deaths (15%) of women in the United States^1^. Many novel chemotherapeutic, hormone-based, and combination drug regimens have been tested in breast cancer, however, survival of advanced aggressive cases is still poor, especially in triple-negative breast cancers (TNBCs), which currently makes up 10–20% of all breast cancer cases^2^,^3^. These types of breast cancer do not express important potential therapeutic targets including receptors for estrogen, progesterone and HER2, resulting in poor overall survival and distant recurrence-free survival. Almost 70% of deaths in TNBCs occurred in the first 5 years following diagnosis compared with only 44% of deaths in other types of breast cancers^3^.

Although adjuvant chemotherapy, as well as radiation, hormonal, and targeted therapy, have all been used to treat different stages of breast cancer, chemotherapy continues to be the major therapeutic option for patients with metastatic disease^4^. While many cytotoxic drugs (including doxorubicin, vinorelbine, gemcitabine, *nab*-paclitaxel, pemetrexed, platinum salts, etoposide, and irinotecan) have been developed for the treatment of metastatic breast cancer, the response rates for these chemotherapeutic agents are usually poor, and the frequency at which patients develop drug-resistance remains high^5^. Thus, the need for combination therapies that can improve the efficacy of current treatment regimens in breast cancer is highly evident.

Despite being the frontline therapy for numerous cancer types, chemotherapy suffers from a general lack of selective toxicity, resulting in a narrow therapeutic index that often compromises clinical prognosis. This is largely due to limitations associated with pharmacokinetics and solid tumors having a high interstitial fluid pressure, which limits bio-distribution and penetration of drugs^6^,^7^. Previous work has shown that the amount of drug accumulated in normal viscera is ~10- to 20-fold higher than that in the same weight of tumor site^8^,^9^, and that many anticancer drugs are not able to penetrate more than 40–50 μm (equivalent to the combined diameter of 3–5 cells) from the vasculature^10^,^11^. These defects often lead to an incomplete tumor response, drug resistance, and ultimately, therapeutic failure^12^.

We have recently reported that non-invasive radio frequency (RF) fields may have the potential to overcome chemotherapeutic drug diffusive transport limitations in the treatment of both pancreatic ductal adenocarcinoma (PDAC) and hepatocellular carcinoma (HCC)^13^,^14^,^15^. Specific effects observed with cancer cells *in vitro* include changes to cell-cell adhesion, elasticity and morphology^16^, which offer the potential to affect drug transport, distribution, and accumulation within tumor tissue. Additionally, we have shown that RF exposure increased interstitial transport and perfusion of fluorescent probes from tumor-associated vasculature^17^. However, deep-seated tumors such as PDAC and HCC have been historically difficult to treat due to issues regarding the fast heating of subcutaneous fat and dermis layers^18^. Additionally, the geometry of the target tissue in the RF field determines heating rates^19^, which has been confirmed by our previous studies, which found that protrusive geometries heat more efficiently when compared to geometries that are flat (data unpublished). Heating can also be dampened by large blood heat sinks if tumors are located near major vessels^20^. Therefore, we propose that RF field therapy may be a potential treatment option for patients with breast cancers, as this malignancy has higher levels of fat proximal to, and inside the tumor, and possesses an advantageous geometry both of which can greatly enhance tumor heating. Additionally, the breast provides easy access to imaging modalities such as ultrasound so that tumor position, alterations in blood flow, tumor structure and surrounding healthy tissue can be monitored in real-time.

The objective of this work is to optimize the short-term treatment schedules of drug and thermal dose administration to breast tumors with the aim to maximize drug localization within the tumor loci. Given the large variety of parameters that must be investigated to optimize drug uptake (i.e. time, temperature, injection time, rise-time, etc.) traditional mass spectroscopy analysis techniques such as liquid chromatography mass spec (LC-MS) and high performance liquid chromatography (HPLC) are either too expensive and/or too lengthy to give rapid, repeatable results. This led us to develop an optical analog method whereby we replace our drug in question with an optical fluorescent dye, which closely mimics the *in vivo* properties of the systemically administered drug. The accumulation of this fluorophore could then be quantified through the use of standard photo spectroscopy techniques (such as a common-use plate reader), after extracting the fluorophore from the tumor.

In this work, Alexa-Albumin 647 nm (Thermofisher, USA) (Alexa647) was chosen as the optical drug analog to nab-paclitaxel (one of the most used anti-breast cancer drugs). These compounds are similar in molecular weight (MW nab-paclitaxel = 853.918 g/mol, MW Alexa647 = 1155.06 g/mol) and blood pharmacokinetics (Figure S1)^21^. It is also postulated that albumin is the major covariate for the kinetics of both compounds through the vascular system and in the tumor microenvironment^21^. We hypothesized that under the right optimized conditions, pre-exposure to heat via RF field therapy in a murine 4T1 breast cancer model will alter vascular dynamics within the tumor environment and will therefore lead to enhanced fluorophore (and therefore drug) accumulation and efficacy. We assumed that dye and drug tumor loading can be similar based on the physical mechanism of vessel response and presence of albumin as a pharmacokinetic “driver factor”.

## Materials and Methods

### Ethic statement and general mice conditions

All experiments were performed after approval of the Institutional Animal Care and Use Committee (IACUC) of the Baylor Collage of Medicine (No. AN-6448) and followed established protocols. Female Balb/c Nude mice were housed in standard temperature and lighting conditions with free access to food and water. All experiments (including heat delivery and imaging) were performed under isofluorene anesthesia (0.7–2.5% isofluorene in medical air). During anesthesia, the mouse condition was monitored with rectal probes and the breathing frequency was established around 1 Hz, after which the animal was kept in a pre-warmed recovery chamber.

### Tumor model

4T1 cells purchased from American Type Cell Culture (ATCC; Rockville, MD) were cultured in Roswell Park Memorial Institute (RPMI) 1640 media supplemented with 10% fetal bovine serum (FBS). Cells were maintained at 37 °C in a humidified atmosphere with 5% CO_2_. 10^5^ 4T1 breast cancer cells suspended in base medium were injected into the left inguinal gland (27G needle, 50μm) to initiate orthotropic 4T1 breast tumors. Treatment was started approximately 12–14 days after cells were injected when tumors had reached 244±68 mm^3^ in volume.

### Radiofrequency field exposure

**Figure 1:**
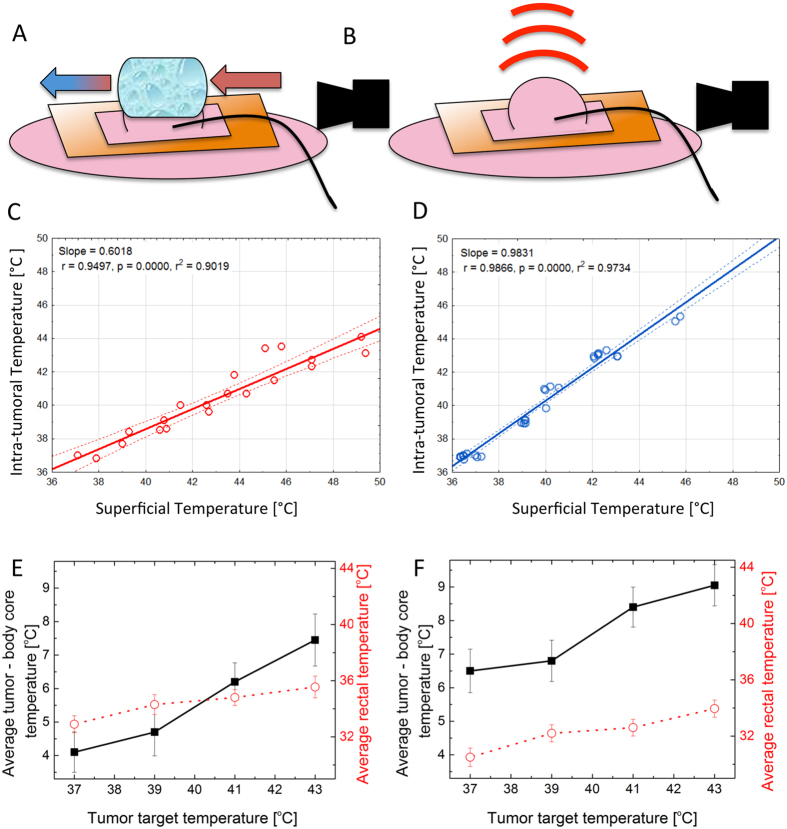
Comparison of treatment protocols for HC (left) and RF (right). (**A**) Schematic depicting HC experimental set-up with mouse. (**B**) Depicts RF experimental set-up with mouse. (Schematic includes orange copper blanket, pink mouse and tumor pulled through square cut into copper blanket, black curved line represents optical thermal probe and solid black shape represents IR camera). (**C**) The relationship between superficial and intra-tumoral temperature in HC. (**D**) The relationship between superficial and intra-tumoral temperature in RF heated tumors (Solid lines represent a linear fit and doted lines represent a 95% confidence interval). (**E**) Mean heat differential between tumor temperature and mouse body (rectal) (left Y axes and square ± SD) temperature (°C) during HC treatment and average rectal temperature (°C), (right Y, dots ± SD). (**F**) Mean heat differential between tumor temperature and mouse body (rectal) temperature (°C) during RF treatment (left Y axes and square ± SD) and average rectal temperature (°C), (right Y, dots ± SD). (Table S1 in supplementary outlines the temperatures of the water bath for various tumoral temperatures and some issues arising during heating regimens).

Mice were subjected to high-intensity (~90 kV/m) 13.56 MHz RF fields at various powers (0–1000 W) to administer a bi-phasic thermal dose that includes a ‘ramp up’ phase from 37 °C to target temperature (39 °C, 41 °C, 43 °C) and a second phase which involves a steady state target temperature (39 °C, 41 °C, 43 °C) for 10 or 30 mins. Non-RF heating temperature controls (HC) consisted of the tumor skin surface being heated with heated water from a water bath running through a pipe in contact with the tumor (Fig. 1). No heat controls (NHC), maintaining core body temperature of mouse to similar value as during RF and HC mice were also included. Temperature was measured using fiber-optic thermal probes and an IR-camera. Before the experimental procedure, the IR camera and fiber optic probes were calibrated using a water-bath (data not shown) to ensure accurate thermometry when comparing the tumor skin surface versus the intra-tumoral temperatures.

### Ultrasound imaging

An Edge^TM^ ultrasound system was used to determine blood flow in the tumors before and after treatment. The HFL38x transducer (13–6 MHz), which is recommended for use with human breast and vascular imaging according to the manufacturer (SonoSite, USA), was used in Doppler and Pulsed Wave modes to calculate blood velocity [cm/s] in the tumor with the fixed gate size (1 mm) during whole imaging procedure.

The VisualSonic^®^ Vevo2100 ultrasound imager with MS550 transducer (32–56 MHz) was used to investigate tumor size and shape before and after treatment. The B-mode was used to study tumor morphology and application of 3D-motor allowed us to perform a 3D reconstruction of 4T1 tumors.

### Dye uptake and extraction

The treatment schedules delivered to the mice and experimental procedures relating to the extraction and quantification blood lifetime, bio-distribution and intra-tumoral localization of fluorescent dye were as follows. Mice with visible tumors were randomized into three groups. Group 1 (RF): group of mice to receive RF hyperthermia for 10, 20 or 30 mins at 37 °C, 39 °C, 41 °C or 43 °C. Group 2 (HC): Heating Control mice received 10 or 30 mins contact hyperthermia treatment, heated to 37 °C, 39 °C, 41 °C and 43 °C via a water balloon placed on the tumor with pre-warmed water being flowed through the device. Group 3 (NHC): Non-Heating Control mice whose core body temperature (measured as rectal temperature) was kept at temperatures similar as possible to treatment mice for 10 and 30 mins. As quickly as possible, (up to 2 minutes) after treatment Alexa647 was administered via an intravenous tail vein injection (10 mg/kg of BW). Twenty-four hours after injection, the liver, tumor, and blood were collected from the mice and the tissues were homogenized to calculate and analyze dye accumulation.

The rationale to select 24 h time-point for organs collection after dye injection was made based on blood pharmacokinetic analysis of Alexa647. We selected this time point to be sure that all dye had cleared the blood, therefore measurements of dye uptake into the tumor were not affected by background dye content in intra-tumoral blood supply (Figure S1). AlexaFluor extraction from the tissue was performed according to the following steps: (1) collected tissue was stored on ice up to 4 h, after which tissue flash freezing in liquid nitrogen was performed (we validated [data not shown] that the storage time and flash freezing did not affect the fluorescence in a non-predictable way), (2) homogenization in cryoPREP™ Extraction System in ethylene glycol (EG), (3) incubation on ice for 10 min with shaking every 2–3 minutes, (4) 5 min of sample sonication, (5) centrifugation at 4,000 rpm for 5 min at 4 °C, (6) supernatant collection – first fraction of tissue extraction into the EG contained the highest amount of dye, (7) re-suspension of the pellet in EG, (8) sonication, incubation, and centrifugation according to above parameters, (9) second supernatant collection, (10) pellet re-suspension in EG, (11) sonication, incubation, centrifugation, and (12) third supernatant collection.

All sample processing including supernatant collection and storage was performed on ice. The ‘three step dye extraction protocol’ was sufficient to obtain more then 95% of the dye from the tissue according to the described protocol (additional extraction in EG is not necessary). Alexa647 content in tumor and blood was quantified based on fluorescent intensity in each supernatant using a CLARIOstar fluorescent plate reader (BMG LABTECH, USA). The calibration curve was designed independently for each type of supernatant (1^st^, 2^nd^, 3^rd^ supernatants contain different concentration of dye so adjustment of calibration curve was necessary to provide a reading in the linear part of curve). Fluorescence spectra were analyzed using MARS data analysis software (BMG LABTECH, USA). For each sample, the maximum fluorescence signal was calculated in the same range of wavelength with background fluorescence subtracted. To determine total dye accumulation inside the tumor tissue the total amount [mg] of dye was calculated (all three fractions of extraction included) and represented in relation to tumor weight. To eliminate the influence of small difference of injected dose per mice (related to syringe scale and effectives of particular injection) each intra-tumoral dye concentration was represented as a percentage of injected Alexa647 dose into particular mouse.

### Experimental design and data analysis

The software STATISTICA12^®^ was used to perform data analysis and experimental design. All experiments were projected with use of Design of Experiments (DOE) module of STATISTICA12^®^ with advance factorial planning: each independent factor (e.g. treatment type, temperature and time) were randomized independently. To determine the statistical significance of presented data the ANOVA module was used. To perform the data fitting the standard database of mathematical function was used with 0.95 confidence border marked with the dotted line. Equation 1 was used to determination the heterogeneity of dye accumulation as a function of temperature and as a function of time, where V = heterogeneity, S.D. = standard deviation and A = average of dye accumulation between groups.


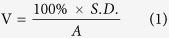


## Results and Discussion

First, we investigated the benefits of using RF electric fields to deliver heat to tissues compared to contact heating via circulated warm water. After testing a few prototypes, we engineered a heating control (HC) device where pre-warmed water flowed through a water bag that was in direct contact with the tumor skin surface (Fig. 1A). Figure 1B depicts the RF mouse set-up. In both HC and RF groups, a fiber optic thermal probe was placed on the surface and another was placed in the center of the tumor. Additionally, the IR camera was used to monitor tumor and skin surface temperature during the heating for further correlation with fiber optic probes (in the next experimental steps this allow for non-invasive tumor temperature monitoring). Rectal probes were also used to ensure that core temperatures in mice heated with HC and RF were kept as consistent as possible during treatment (33.5 + /− 2.5 °C). We observed that RF exposure produced more consistent heating throughout the tumor tissue, whereas the direct contact HC induced a more significant heat differential across the tumor tissue (Fig. 1C and D). Heating consistency and safety via RF was increased further by the addition of a copper blanket during RF field exposure as it enabled more localized and controlled RF heating due to shielding other mouse body parts from the radiating RF electric field. Homogenous heating throughout tumor tissue is significant in two main ways: (i) consistent heating through a tumor ensures whole tumor hyperthermia and means cancer cells, such as cancer stem cells, in various tumor microenvironment niches do not escape RF hyperthermia. Additionally, RF can be used to heat tumors non-invasively. (ii) Intra-tumoral temperatures are more easily predicted from tumor surface temperature readings. This is significant because the insertion of an intra-tumoral probe alters the tumor microenvironment and may have influence over drug accumulation or induce inflammatory responses.

We further investigated the difference between tumor temperature and core body temperature to explore how heat is localized and distributed systemically by RF and HC heating. This is an important consideration as excessive increases in core body temperature due to this form of heating can lead to complications for the animal under investigation, especially if small rodents are being utilized. For instance, increases in body core temperature can lead to alterations in metabolic heat production, homeothermy, blood perfusion, thermoregulation, immunologic and physiologic effects^22^. Altering these biological parameters in the animal may not only be detrimental to the rodent under investigation but also introduces a large variety of variables that may skew scientific results.

Figures 1E and F shows that the difference between tumor and body core temperatures is indeed different between the two heating methods. For example: as can be seen in Fig. 1C, using the HC method of heating, for a target tumor temperature of 41 °C (at 30 minute hold time) the superficial temperature needs to be 44.5 °C, which in turn results in a 6 °C temperature differential (on average) between the tumor and core body temperature. In comparison, with respect to RF heating, a more selective and localized tumor heating profile is visible; with a larger heat differential observed between body temperature and tumor. For example, for 30 min heating at 41 °C target temperature we observed <1 °C difference between intra-tumor and superficial temperature (Fig. 1D) and an 8.5 °C difference between tumor and core-body temperature, which is representative of localized, targeted heating (Fig. 1). This finding, coupled with the more homogeneous heating profile (Fig. 1C,D) suggests that RF is a more *predictable, uniform, reliable* and *safer* method of delivering hyperthermia, non-invasively, to tumors in a 4T1 murine models when compared to the HC method.

Using hyperthermia to increase intra-tumoral drug accumulation is one of the main goals surrounding tumor hyperthermia therapy as it can provide a method for overcoming the limitations of tumor diffusive transport and high intra-tumoral pressures^23^. We have previously shown that RF treatment causes increases in extravasation of nanoparticle-sized molecules from tumor-associated vasculature into the tumor tissue^24^.

To determine the optimal hyperthermia treatment schedule administered to 4T1 tumors we designed an exploratory investigation that considered a range of thermal doses administered via RF or HC mechanisms before drug administration. We used Alexa647 as a drug analog due to the fact that it is an albumin bound molecule (as in *nab*-paclitaxel – albumin bounded paclitaxel). Clinically, *nab*-paclitaxel is used to treat aggressive breast cancer after failure of combination chemotherapy for metastatic disease or relapse within 6 months of adjuvant chemotherapy^25^. Alexa647 is non-toxic in tested doses and can emit very characteristic and strong fluorescence, enabling for accurate and easy dosimetry analysis of the tumor tissue and various healthy organs.

**Figure 2:**
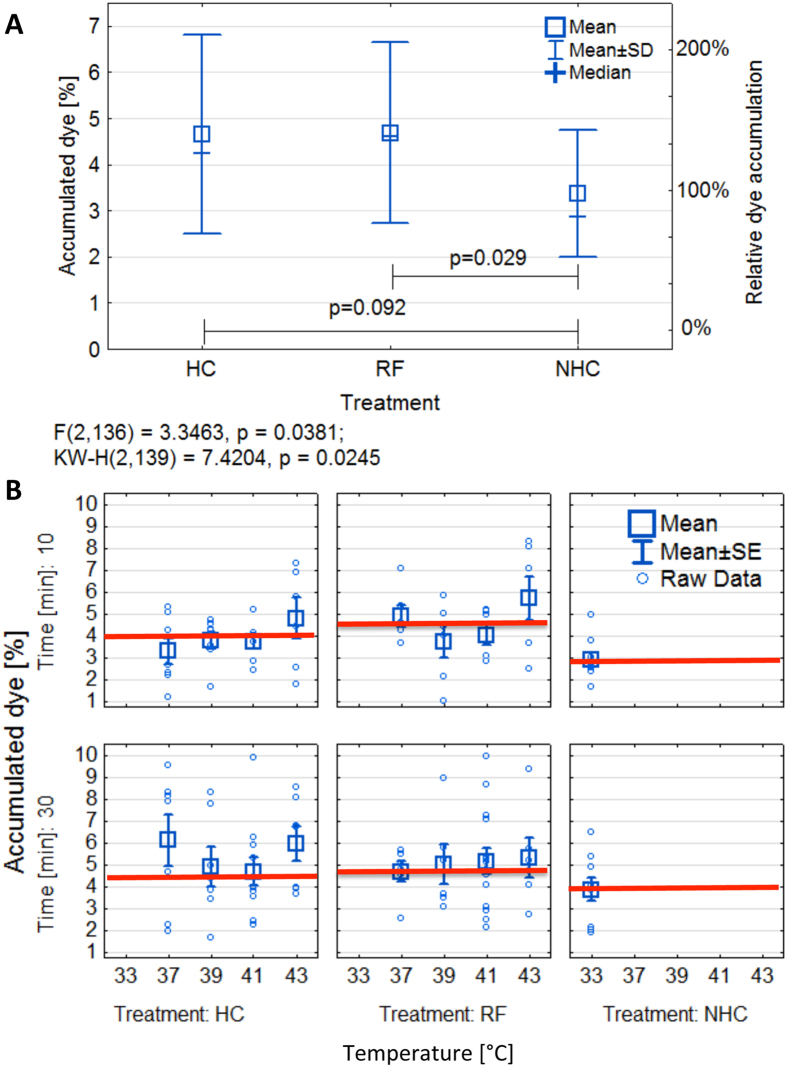
Accumulation of injected dye dose in the tumor 24 h after treatment. (**A**) Percentage of injected dye accumulated in tumors after HC, RF and NHC treatment (all thermal doses are grouped together in this analysis). The current ANOVA effect is described under the plot, the error bars represent 95% confidence interval. (**B**) Percentage of injected Alexa647 in the 4T1 tumors in NHC, HC and RF groups in individual holding times (rows) and temperature (upper X scale). Data is presented as means ± standard errors (SE). Total N in experiment is 139 tumors. Average tumor volume 244 ± 68 μL. (Red lines represent median for whole treatment group).

Figure 2 shows the percentage of total injected dye that accumulated in the tumor with various heating groups. The accumulated dye dose was calculated as a percentage of total injected dose (per particular mouse) in relation to the dose found in the whole tumor mass (the combination of three extraction phases) according to Materials and Methods information. When all thermal doses (i.e. 37 °C, 39 °C, 41 °C and 43 °C) are considered together in each group, both HC and RF treated groups displayed an increased inter-tumoral accumulation of dye when compared to the NHC group (Fig. 2A). We observed the greatest significance between the RF and NHC groups (p = 0.029) (Table S2). The average uptake of dye in the NHC mice is approximately 3.5%, HC is 4.5%, and the RF is 4.8%. Based on this data hyperthermia enhances dye delivery into the tumor tissue by approximately 1.5% of total injected dose, which is translated to an increase of 40% when compared solely to the NHC group.

Additionally, RF displayed a more homogeneous dye accumulation profile across all thermal doses when compared to HC (approximately 35% vs 45% of inter-tumoral heterogeneity of dye distribution), which is most likely due to RF’s capability to promote more uniform heating of the tumor tissue. Figure S2 in the Supplementary Information section provides information regarding inter-tumoral heterogeneity of dye accumulation after treatment calculated as relation between mean accumulation and group standard deviation (Eq. 1). Once again, this is an important consideration as homogeneous chemotherapeutic delivery across tumor tissue is therapeutically and clinically advantageous. Furthermore, we observed significant differences in dye uptake even among mice of the same treatment group, which demonstrates the extent to which tumor heterogeneity plays a detrimental role for predicting drug delivery in solid tumors.

When the groups are split further into individual thermal doses within treatment groups, then the heterogeneity of tumor dye uptake after hyperthermia becomes even more apparent (Fig. 2B). The tumor response and thermal dose relationship with regards to increases or decreases in influx of dye into the tumor tissue is more difficult to predict. However, the relationship between dye accumulation and temperature is clinically useful to investigate the optimal “thermal dose” needed to induce maximal uptake of dye. Biological and tumoral heterogeneity, especially very heterogeneous and pathological tumor vasculature systems, likely play a role in the big variation of dye uptake observed across individual groups and between thermal doses across a particular heating method. This means that statistical significance is difficult to achieve when results are split into individual thermal dose groups within HC, NHC and RF treatment cohorts. However, based on these findings, as well as the issues listed in Table S1, we decided to further explore the RF hyperthermia effect on dye uptake using 41 °C for 30 min regimen.

This particular RF thermal dose regimen resulted in a 33.3% increase of the total intravenously administered dye being delivered to the tumor when compared to the NHC group. This represents a substantial increase in tumor dye delivery and is a particularly relevant finding, as it is well known that only a very small fraction of total chemotherapeutic dose without adjuvant therapy localizes in the tumor^1^,^2^,^8^,^9^,^10^,^11^. The decision to further explore the 41 °C for 30 min thermal dose regimen was not only based on the increases observed in dye delivery to the tumor but also on various other factors such as safety issues for the mice; in particular the ability to keep their core temperature relatively consistent and to limit the risk of skin burns (Table S1). The NHC at 30 minutes was also chosen to give a more comparable control with regards to the temporal element in the treatment schedule of the mice.

**Figure 3:**
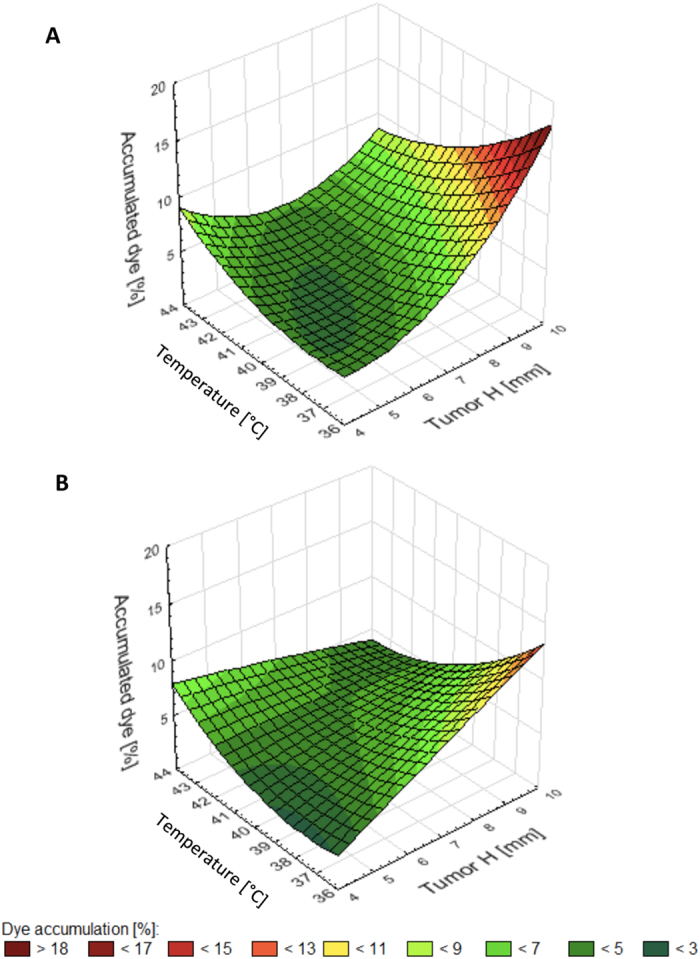
Relation between dye accumulation and various experimental factors. (**A**) Simulated/fitted relation between dye accumulation [% of injected dose], tumor height [mm] and temperature during RF. (**B**) Simulated/fitted relation between dye accumulation [% of injected dose], tumor height [mm] and temperature during 30 min of HC.

Next, we sought to investigate the factors influencing the degree of dye accumulation heterogeneity seen in RF and HC groups. Figure 3 shows the relation between dye accumulation, temperature and geometric characteristics of the tumor. The relationship between the position of the transmitting and receiving electrodes of the RF field and the site undergoing thermal exposure, including its geometry is known to be a clinically relevant factor in RF hyperthermia^26^. Therefore investigating the relationship between tumor geometrical factors (such as height, width, and general 3D heterogeneity) and associated RF-induced heating may provide mechanistic insight into improving non-invasive RF heating efficacy in malignant tissues^27^. Height is of particular importance as the closer the tumor is to the transmitting head the more intense the electric field on the tumor and the greater the heating profile. In addition to RF heating efficacy being dependent on geometrical factors, the tumor geometry may also play a role in how tumors release heat to the surrounding environment during their exposure to RF fields. This has more relevance in a breast or subcutaneous mouse model where the tumor to skin surface is higher than orthotopic murine models. Heterogeneity in tumor vascularization and various intra-tumoral histopathological features, such as stroma constituents and fat deposits (fat heats especially well in RF fields at 13.56 MHz) further obscure any significance. Figure 3A and B show the fitted relation between dye accumulation [% of injected dose], tumor height [mm] and temperature during RF (left) and HC (right) 30 min heating. The simple comparison between RF and HC clearly shows the large role the tumor geometry plays in RF hyperthermia drug accumulation efficacy regardless of what thermal dose is administered. (Statistics are displayed in the Supplementary, Table S2 and selected data displayed in Figure S3).

**Figure 4:**
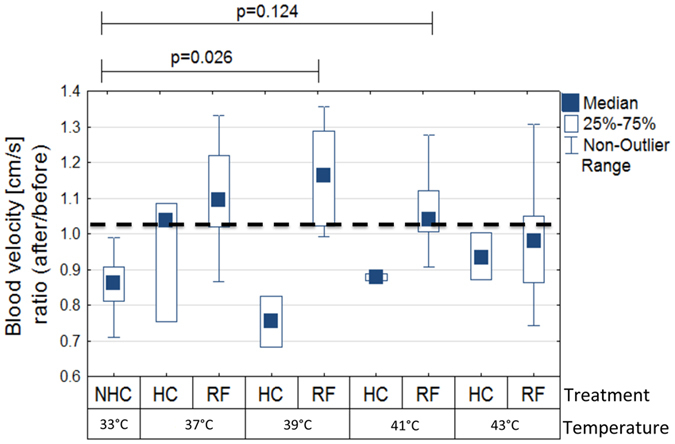
Effects of hyperthermia on tumor fluid dynamics. The blood velocity for various treatments (ratio of before to after treatment, boxes represent the mean and bars represent the standard deviation).

Fluid dynamics, such as blood flow inside tumors represents another source of heterogeneity that dictates the accumulation of drug molecules into tumor loci. Tumor fluid dynamics differs drastically from the dynamics observed in normal tissue due to alterations in both morphology and structure of tumor-associated vasculature. Tumor vessels show highly pathological features on molecular and structural level of vasculature network^28^,^29^. There are also abnormalities of the vessel walls, and geometric and chemical complexity of the interstitium around tumors^30^,^31^,^32^ and microvascular network architecture and hemodynamics exhibit a high degree of heterogeneity^33^. Therefore blood flow inside these networks is highly unstable and prone to alterations, even changes in direction^34^. The leakage of blood plasma leads to an increase in the interstitial pressure, causing vessel occlusion and acute hypoxia. Diffusive processes largely characterize the transport of drugs in the tumor microenvironment, as pressure gradients near tumors are minimal, except at the tumor margins. We postulate that RF-hyperthermia can affect the tumor vessels function for short term (till around 30 min after the treatment) and by tissue thermal expansion change intratumoral pressure – in the result we expected increase of fluid dynamic as blood velocity in the tumor tissue (Fig. 4). Blood velocity is an important consideration as it has been previously shown that hyperthermia alters blood velocity in biological tissues^35^ and hence will represent one of the mechanisms of altered dye accumulation in tumor loci due to hyperthermia. We are able to prove that only after RF treatment, especially after 39 and 41 °C tumor temperature, the average blood velocity inside the tumor is increasing for around 15% (in comparison to the situation before the treatment).

## Conclusion

Interactions of radio waves with biological tissues are currently being investigated as a therapeutic platform for non-invasive cancer hyperthermia therapy. RF delivers thermal energy into tissues, which increases intra-tumoral drug perfusion and blood-flow. Herein, we describe an optical-based method to optimize the short-term treatment schedules of drug and hyperthermia administration in a 4T1 breast cancer model via RF, with the aim of maximizing drug localization and homogenous distribution within the tumor microenvironment. This method, based on the analysis of fluorescent dyes localized into the tumor, is more time-, cost- and resource-efficient, when compared to current analytical methods for tumor-targeting drug analysis such as HPLC and LC-MS. Alexa-Albumin 647 nm fluorphore was chosen as a surrogate for nab-paclitaxel based on its similar molecular weight and albumin driven pharmacokinetics. We found that RF hyperthermia induced a 30–40% increase in Alexa-Albumin into the tumor micro-environment 24 h after treatment when compared to non-heat treated mice. Additionally, we showed that the non-invasive RF method of delivering hyperthermia to tumors was more localized and uniform across the tumor mass when compared to other direct-contact methods of heating. Lastly, we provided insight into some of the factors that influence the delivery of RF hyperthermia to tumors.

## Additional Information

**How to cite this article**: Ware, M. J. *et al*. Optimizing non-invasive radiofrequency hyperthermia treatment for improving drug delivery in 4T1 mouse breast cancer model. *Sci. Rep.* **7**, 43961; doi: 10.1038/srep43961 (2017).

**Publisher's note:** Springer Nature remains neutral with regard to jurisdictional claims in published maps and institutional affiliations.

## Supplementary Material

Supplementary Information
