# Supplementary material for: Optimizing non-invasive radiofrequency hyperthermia treatment for improving drug delivery in 4T1 mouse breast cancer model
Source: Sci Rep. 2017 Mar 13;7:43961. doi: 10.1038/srep43961 (PMC5347121; doi:10.1038/srep43961)
Supplement: Supplementary Information [file srep43961-s1.pdf]

Supplementary

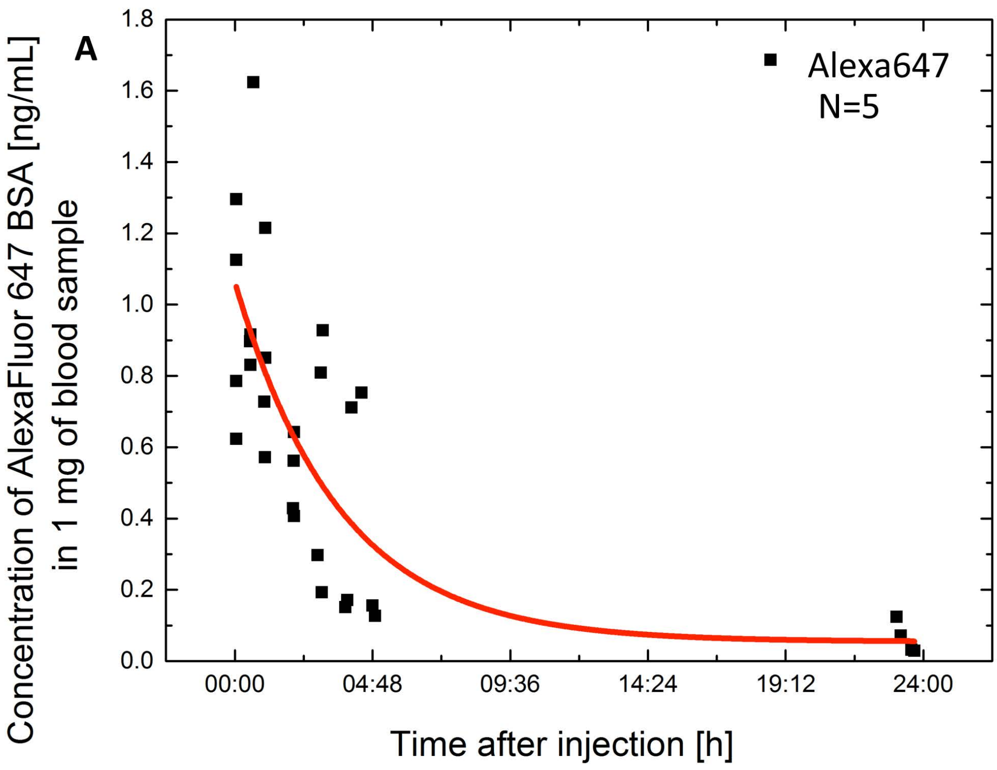

Figure S1: Pharmacokinetics of Alexa647 between 0-24h.

	HC		RF
Target tumor temp. [°C]	Aim for superficial tumor temp. [°C]	Water bath temp. [°C]	Aim for superficial tumor temp. [°C]
37	37-38	39-40	37
39	41	44-45	39
41	44-45	48	41
43	48	51-53	43
Side effects:	• Burns on tumor surface<br>• Big heat differential<br>• Surrounding tissue heating		• Random burns

Table S1: Table outlining the temperatures and some issues arising during heating regimens.

		Temperature vs …				Irradiation time vs …	
		37°C	39°C	41°C	43°C	10 min	30 min
NHC vs RF	0.029	0.57	1	1	0.23	0.15	1
NHC vs HC	0.092	1	1	1	0.27	1	1
HC vs RF	1.00	1	1	1	1	1	1

Table S2: The p values for significance between treatment, temperature and time influence during RF exposure (n=139).

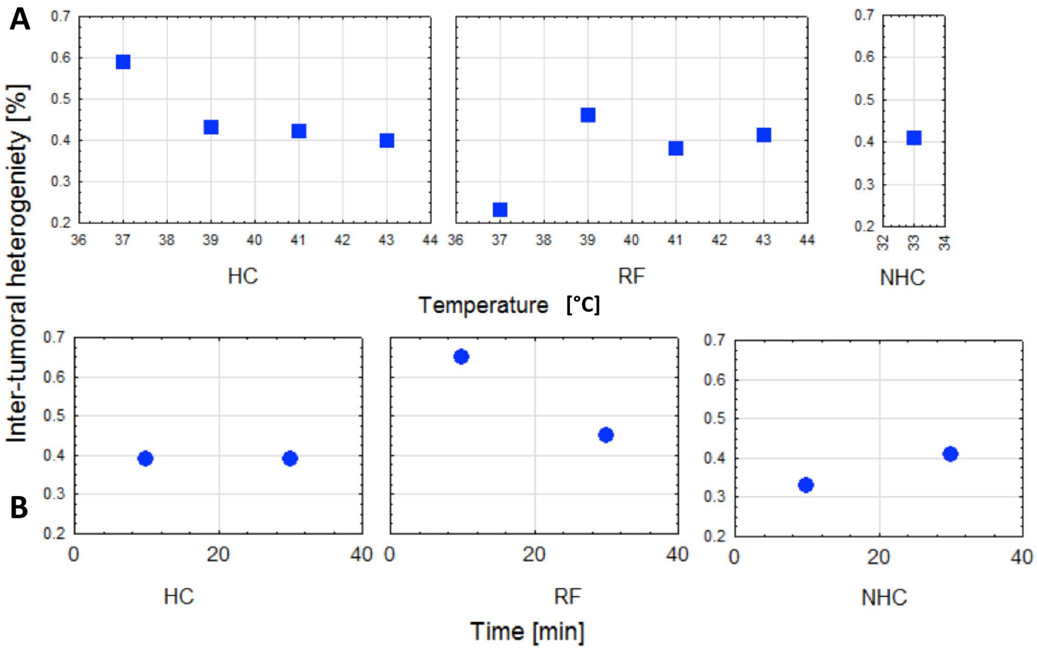

Figure S2: Inter-tumoral heterogeneity of dye accumulation after treatment calculated as a relation between mean accumulation and group standard deviation. A) Inter-tumoral heterogeneity of dye accumulation after treatment as a function of temperature. B Inter-tumoral heterogeneity of dye accumulation after treatment as a function of time.

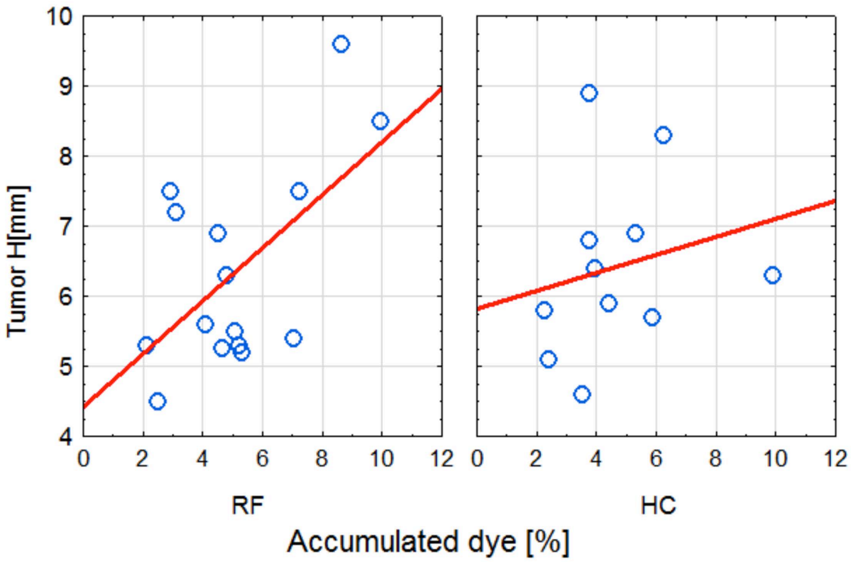

Figure S3: Selected example of correlation between tumor height and dye accumulation (41° C for 30 min). (RF, slope = 0.3796, r = 0.5911, p=0.0203, r^2 = 0.3494. HC, slope = 0.1281, r = 0.2154, p=0.5247, r^2 = 0.0464)
